# Roles of Eleven Key Enzymes in Sweet Value and Soluble Sugar Component Content During Mango Development

**DOI:** 10.3390/ijms27167486

**Published:** 2026-08-21

**Authors:** Li Li, Zisong Wang, Weiming Li, Xiang Li, Kaiyi Zou, Xiaofen Xie, Chenxing Liu, Yanke Wu, Jiehuan Chen, Guodi Huang, Caihua Liu

**Affiliations:** 1 Guangxi Key Laboratory of Biology for Mango, College of Agriculture and Food Engineering, Baise University, Baise 533000, China; liliaaa666@163.com (L.L.);; 2 Guangxi Engineering Research Center of Green and Efficient Development for Mango Industry, Guangxi Subtropical Crops Research Institute, Guangxi Academy of Agricultural Sciences, Nanning 530001, China; 3 School of Marine Sciences and Biotechnology, Guangxi Minzu University, Nanning 530008, China; 4 College of Mathematics and Computer, Guangdong Ocean University, Zhanjiang 524088, China

**Keywords:** mango, carbohydrate, metabolic enzyme activity, relational mathematical model

## Abstract

Mango fruits are popular for their flavor. Sugar, a key component of fruit nutrition and flavor, determines fruit quality via its composition and content. While many studies explore molecular regulatory pathways of sugar metabolism in various fruits, large-scale physiological screening of related enzymes via advanced mathematical methods is largely neglected, and the physiological mechanism of sugar accumulation in mango remains unclear. This study analyzed glucose, fructose, sucrose and starch contents, and the activities of 11 sugar metabolism-related enzymes (adenosine diphosphate glucose pyrophosphorylase (AGP); sucrose synthase (SS); sucrose phosphate synthase (SPS); protein kinase (PK); starch debranching enzyme (DBE); phosphoglucomutase (PGM); α-amylase; β-amylase; isoamylase (ISA); sucrose invertase (INV); acid invertase (AI)) from fruit growth to post-ripening stages of mango cultivar ‘Renong No.1’, determined the correlations among soluble sugar, starch and enzyme activities, and constructed mathematical models of inter-group relationships using R language. The results showed that glucose, fructose and starch accumulated during fruit growth, while sucrose accumulated during post-ripening. Fructose content was significantly positively correlated with AGP, α-amylase, AI, β-amylase and SPS activities; so was glucose content and AGP activity; sucrose content and AGP, AI, PK, α-amylase, ISA, INV and SPS activities; and sweetness value and AGP, α-amylase, AI, SPS, SS and β-amylase activities. AI, AGP and α-amylase were identified as the key enzymes influencing sweetness value. Ultimately, a new visualizing and digitizing statistical approach for roles of eleven key enzymes in sweet value and soluble sugar component content during mango development was shown.

## 1. Introduction

Mango (*Mangifera indica* L., Anacardiaceae) is one of the top five tropical fruits in the world (banana, pineapple, litchi, mango and longan) and is well-known as “the king of tropical fruits”. The cultivated area in 2022 was nearly 381,000 ha and the production was about 3.8 million tons across the world [1]. China is one of the major mango producing countries globally. The mango industry is one of the major industries established in several regions of China, such as Hainan, Guangdong, Guangxi, Yunnan, Sichuan, Fujian, and Taiwan provinces [2]. A wide variety of mango resources exist globally, i.e., there are 2360 accessions, with more than 1000 varieties falling within 37 categories. Nearly 900 mango varieties are preserved in China and more than 400 varieties were classified based on characterizations [3]. Sugar, with its diverse composition and content, is the key factor that qualifies fruit quality [4,5]. In general, mango fruits with high sugar content and strong sweet taste are popular in China. As a result, several varieties with appealing appearance are not among the predominant commercial ones because of the low sugar content and sour taste, which in turn also restricts the breeding of new varieties with those good germplasm resources. Therefore, increasing the sugar content of fruits has been a subject of interest among researchers [6,7].

The fruit quality changes considerably during the ripening period [8]. The sugars not only provide energy but also play an essential part in influencing flavor and the nutrients of fruit [9]. Sugar accumulation is influenced by a broad range of factors and processes, such as production, transportation and distribution of photosynthetic products in fruits. Assimilation products that are eventually transported into fruits are determined by their metabolism in fruits [10]. As a powerful metabolic sink, fruits obtain large amount of assimilates from source tissues during the ripening stage [8,11]. Understanding the production, transportation, and distribution of sugar, as well as sugar metabolism and its regulation, would provide insights into the regulation of sugar content and composition in fruits [12]. Consequently, elucidation of the regulatory mechanisms governing genes involved in fruit sugar metabolism, particularly sucrose metabolism, has emerged as a critical research priority in modern fruit tree breeding and quality enhancement [13]. However, despite the proliferation of studies on the molecular regulatory pathways of sugar metabolism in various biological systems such as cherry [14], apricot [15], kiwifruit [13] and microbes [16], large-scale physiological-level screening of relevant enzymes using advanced mathematical approaches has received little attention and has often been neglected.

A few studies have investigated starch metabolism and sucrose accumulation during fruit growth and maturity stages in mango. The sugar accumulated in mango fruits is converted to starch, which is the primary source of carbon for sucrose synthesis and the major form of carbohydrate at the fruit growth stage [5,17]. Starch hydrolysis begins before picking in most mango varieties and sucrose synthesis peaks after picking [18]. As a result, sucrose is the primary component, followed by glucose and fructose at the maturity stage [17,18,19], except for a few varieties in which fructose is the main component [20]. ‘Keitt’ is a special mango variety, where the conversion of starch to soluble sugars only occurs after picking, which makes it a model variety for the study of the conversion of starch to soluble sugars in mango fruits [21,22]. However, the mechanisms of starch–sugar conversion in mango fruits remain unclear because it is difficult to determine fruit maturity and resources are limited [17].

The ability of fruits to obtain assimilates largely depends on the sink strength, which is often determined by the activity of key enzymes related to glucose metabolism [23]. In the glycolysis process, free carbohydrates produced from sucrose and starch are metabolized by different enzymes [24]. In the case of fruit, sugar transformation is closely associated with activities of various sugar metabolism-related enzymes [25]. There are three types of enzymes, including SS (EC2. 4. 1. 13), SPS (EC2. 3. 1. 14), and INV (EC3. 2. 1. 26), associated with sucrose metabolism and sugar accumulation in fruits [26]. Starch in the fruits is hydrolyzed to glucose by amylase, which serves as a substrate for sugar metabolism in mango fruits. Afterward, glucose is converted to fructose by glucose-6-phosphate isomerase. The generated glucose and fructose are synthesized into sucrose by SPS and AI catalyzes the degradation of sucrose to glucose and fructose [27,28]. The enzymes associated with sucrose synthesis during the fruit growth stage of mangoes include INV, AI, SPS, SS, and neutral invertase (NI) [17]. During the ripening stage, SS and SPS activities increase when mango fruits are mature [19,29]. Even so, a new holistic vision and a new statistical method for the related enzymatic activity, the key carbohydrates and the sweet values of mango are still needed.

The studies on starch and sugar (photosynthetic products) contents, starch–sugar conversion in mango fruits, as well as related metabolic enzymes provide crucial data and form a basis for elucidating the physiological and molecular mechanisms underlying sugar metabolism during the growth and maturity stages of mango fruits. However, sugar metabolism in mango fruits is a complex physiological and biochemical process, and the specific induction and response mechanisms involved remain unclear.

Therefore, this study investigated the physiological mechanism underlying sugar metabolism in the mango variety ‘Renong No.1′ during fruit growth and maturity stages. The results of this study could provide crucial information for improving sugar accumulation in mango varieties with low sugar content and facilitate breeding of superior mango varieties.

## 2. Results

### 2.1. Dynamic Changes in TSS, Soluble Sugar, and Starch Contents in Fruits

#### 2.1.1. Fruit in Growth and Physiological Maturity Stages

**Figure 1:**
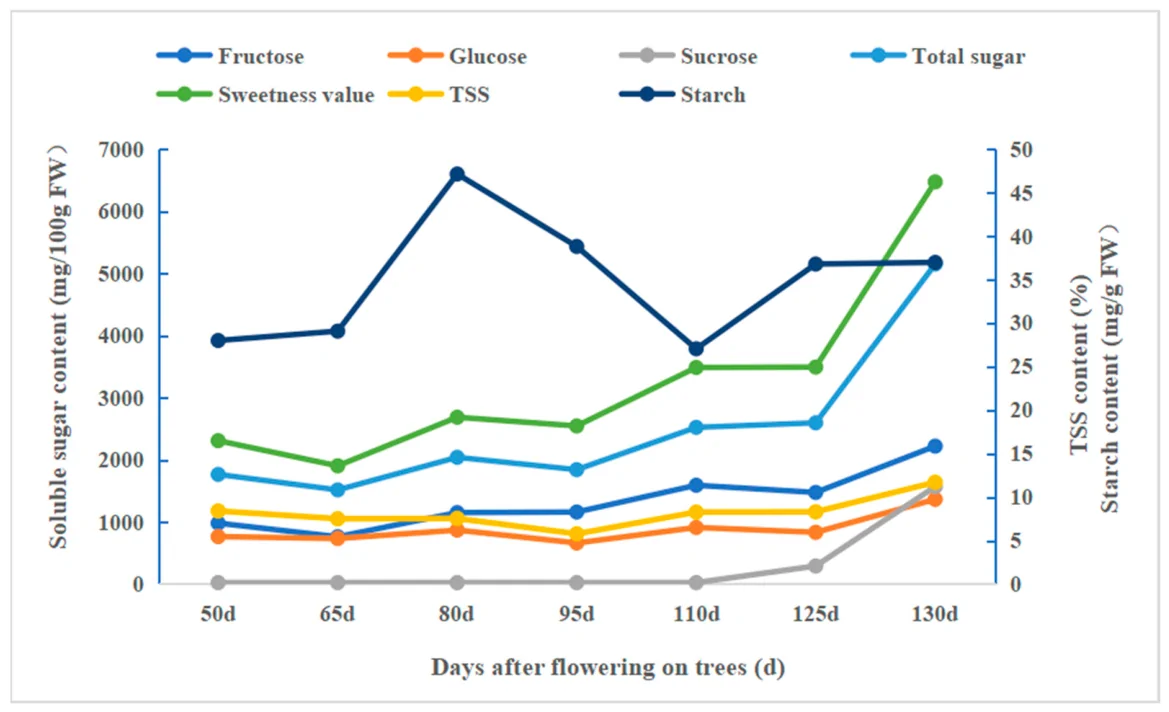
Dynamic changes in total soluble solid, soluble sugar, and starch contents during the growth stage of ‘Renong No. 1’ mango fruits. Note: TSS, total soluble solid.

Trends of total soluble solid (TSS), soluble sugar, and starch contents during the growth stage of ‘Renong No. 1’ fruits are shown in Figure 1. The TSS content initially decreased gradually from 50 days to 95 days after flowering (during the fruit growth stage) and then began to increase gradually until 130 days after flowering. Fructose and glucose contents exhibited a similar trend, with a continuous increase in both contents being observed from 50 days to 130 days after flowering. Sucrose content was low and did not change significantly during the early stage of fruit growth; however, sucrose content increased significantly between 110 and 130 days after flowering (physiological maturity stage, Table 1). Starch content exhibited a fluctuating trend during the sampling period and accumulated rapidly during the fruit expansion period (65–80 days after flowering) and increased significantly to a maximum value of 47.14 mg/g FW (Table 1).

#### 2.1.2. Fruit in On-Tree Ripening Stage

**Figure 2:**
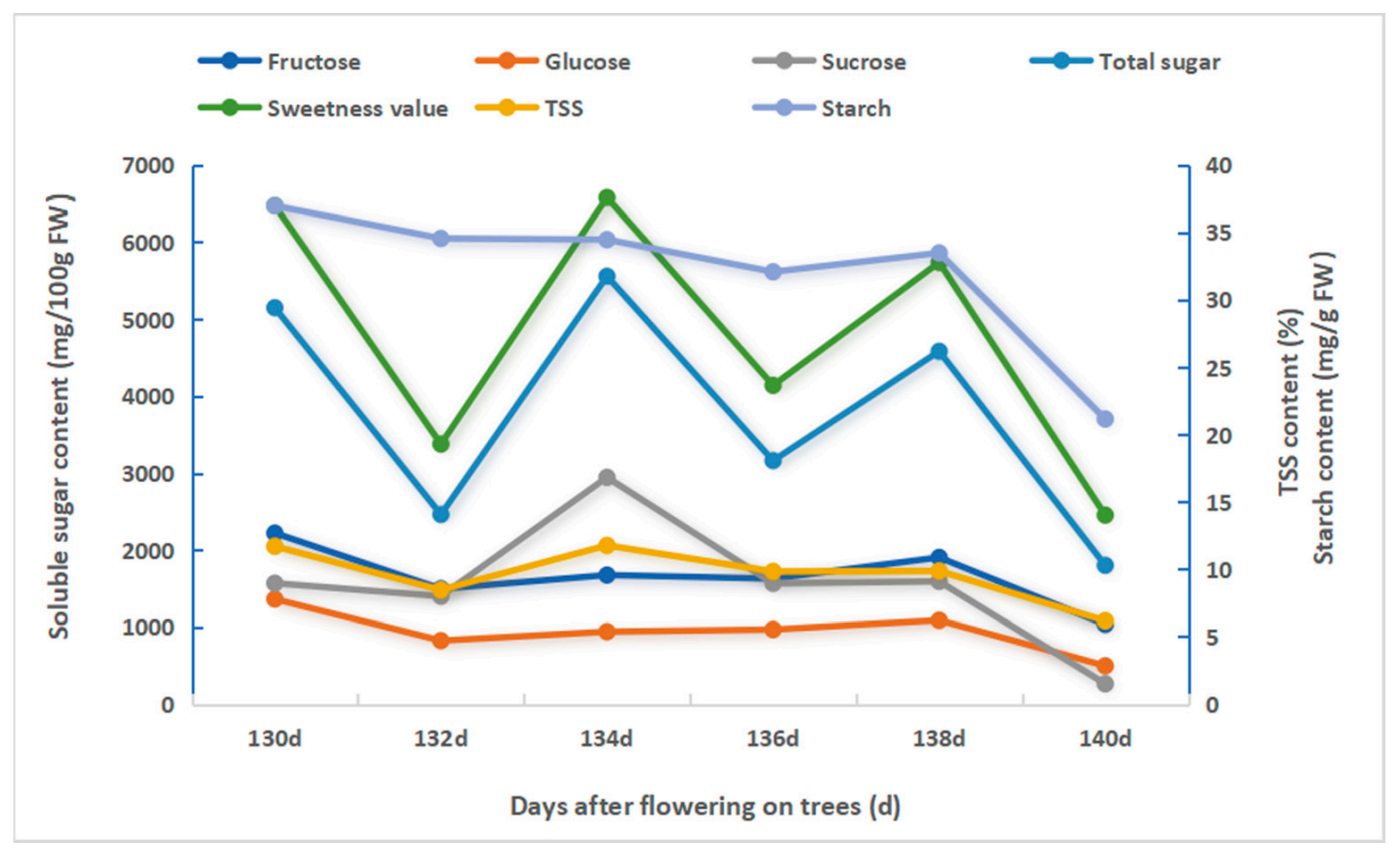
Dynamic changes in total soluble solid, soluble sugar, and starch contents in ‘Renong No. 1’ mango fruits after ripening on the tree. Note: TSS, total soluble solid.

The dynamic changes in sucrose, total sugar contents, and sweetness values of ‘Renong No. 1’ mango fruits left on the trees during the post-ripening stage were highly consistent (Figure 2). The contents of soluble solids, starch, fructose, and glucose were relatively stable and decreased gradually during the post-ripening stage. However, dynamic changes in sucrose, total sugar contents, and sweetness values were similar and decreased progressively. All parameters decreased to the lowest value at 140 days after flowering, suggesting that the fruits undergo severe sugar loss and are prone to rotting (Table 2).

#### 2.1.3. Fruit in Post-Harvest Ripening Stage

**Figure 3:**
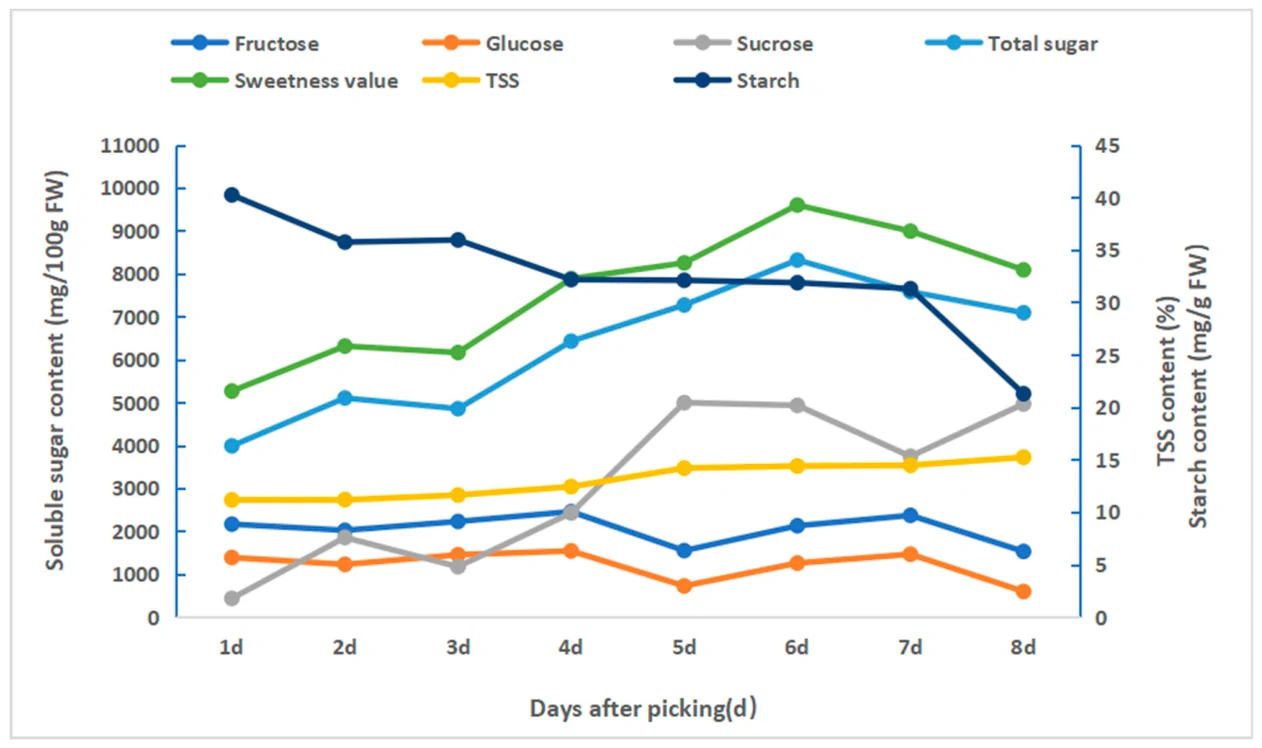
Dynamic changes in total soluble solid, soluble sugar, and starch contents of ‘Renong No. 1’ mango fruits during ripening after picking. Note: TSS, total soluble solid.

The dynamic changes in sweetness values, sucrose, and total sugar contents during post-harvest ripening of ‘Renong No. 1’ mango fruits were different from those of mango fruits left on-tree to ripen. The highest value was observed 5–7 days after harvesting and the highest sucrose content was 4995.03 mg/100 g FW. The dynamic changes in fructose and glucose were similar, with fluctuations throughout the entire experimental period, and peaked at 7 days after harvesting. Starch content and TSS content showed contrasting trends, i.e., the former continuously declined, while the latter increased slightly (Figure 3). Notably, the accumulation trend of sucrose content differed from that of glucose and fructose contents and was contrary to that of starch content. Almost all parameters tend to decline in the last two days, suggesting that the fruits begin to deteriorate (Table 3).

### 2.2. Dynamic Trends of Activities of Enzymes Related to Sugar Metabolism in Fruits

#### 2.2.1. Fruit in Growth and Physiological Maturity Stages

**Figure 4:**
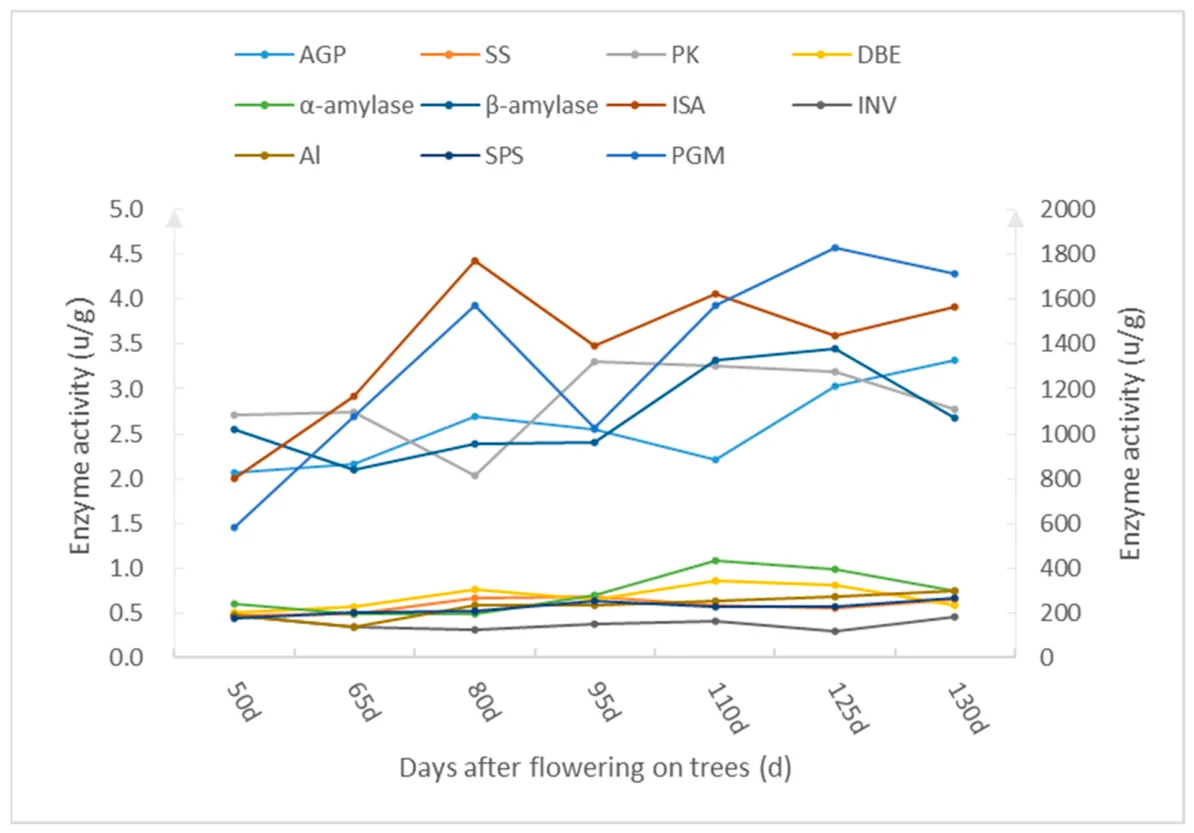
Dynamic changes in the activities of enzymes associated with sugar metabolism in ‘Renong No. 1’ mango fruits during the growth stage. Note: AGP, adenosine diphosphate glucose pyrophosphorylase; SS, sucrose synthase; SPS, sucrose phosphate synthase; PK, protein kinase; DBE, starch debranching enzyme; PGM, phosphoglucomutase; ISA, isoamylase; INV, sucrose invertase; AI, acid invertase.

The dynamic changes in the activities of enzymes associated with sugar metabolism during the fruit growth and physiological maturity stages are shown in Figure 4. According to the results, AI activity decreased during the early growth stage of ‘Renong No. 1’ mango fruits and gradually increased to a maximum value during the subsequent stage. INV activity initially decreased and subsequently increased to a maximum value. Moreover, AI activity was higher than that of INV. The activities of SS and SPS during the whole fruit growth and physiological maturity stages showed an overall upward trend and reached a maximum value at 130 days after flowering. The dynamic changes in α-amylase and β-amylase activities were similar. The activities of α-amylase and β-amylase initially increased gradually to a peak and then decreased gradually afterward. Notably, β-amylase activity was considerably higher than that of α-amylase throughout the growth and physiological maturity stages. AGP activity initially reached a phased peak during fruit expansion, then decreased, and reached a maximum value at the physiological maturity stage. PGM activity was the highest among the enzymes evaluated, with its activity reaching a phased peak during the fruit expansion stage. PK activity decreased during the early fruit growth stage, reached a minimum value during the fruit growth and expansion stages, and then increased gradually back to its initial value. ISA activity initially increased and subsequently fluctuated slightly, while DBE activity initially increased and then decreased to its initial level. Slight changes were observed in PK, DBE, and NI activities when compared to their initial values. The activities of other enzymes associated with sugar metabolism exhibited an upward trend after the early stage of fruit growth.

#### 2.2.2. Fruit in On-Tree Ripening Stage

**Figure 5:**
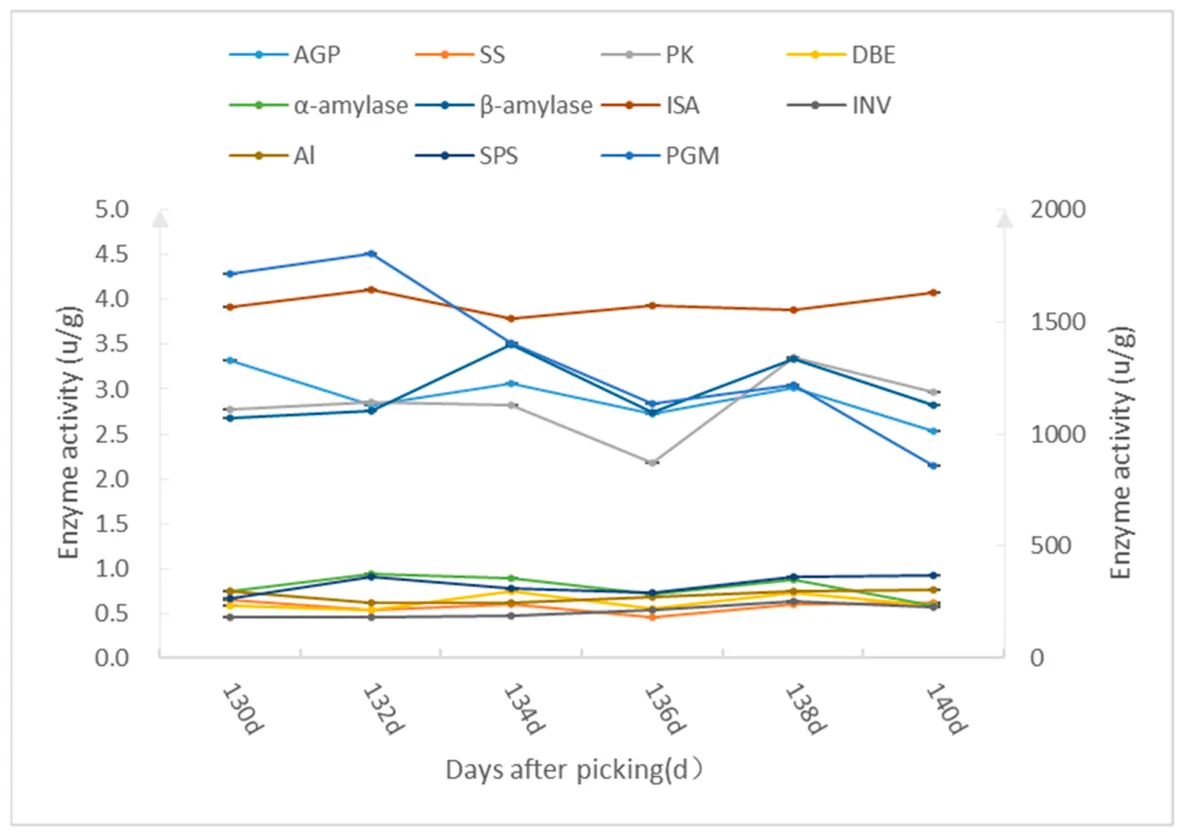
Dynamic changes in the activities of enzymes associated with sugar metabolism in ‘Renong No. 1’ fruits that were left to be ripen on the trees. Note: AGP, adenosine diphosphate glucose pyrophosphorylase; SS, sucrose synthase; SPS, sucrose phosphate synthase; PK, protein kinase; DBE, starch debranching enzyme; PGM, phosphoglucomutase; ISA, isoamylase; INV, sucrose invertase; AI, acid invertase.

The results revealed that AI activity in ‘Renong No. 1’ mango fruits was relatively stable during on-tree ripening, while INV activity exhibited an upward trend. α-amylase activity initially increased and then decreased, while β-amylase activity increased slightly with fluctuations. β-amylase activity was higher than that of α-amylase; SS activity was stable during the entire post-ripening stage; SPS activity initially increased, then decreased but with fluctuations, and finally increased; AGP activity decreased but with fluctuations. PGM activity initially increased slightly, then decreased substantially between 132 and 136 days, and finally decreased gradually at all subsequent stages. PK activity initially increased and subsequently fluctuated; ISA and DBE activities remained stable (Figure 5).

#### 2.2.3. Fruit in Post-Harvest Ripening Stage

**Figure 6:**
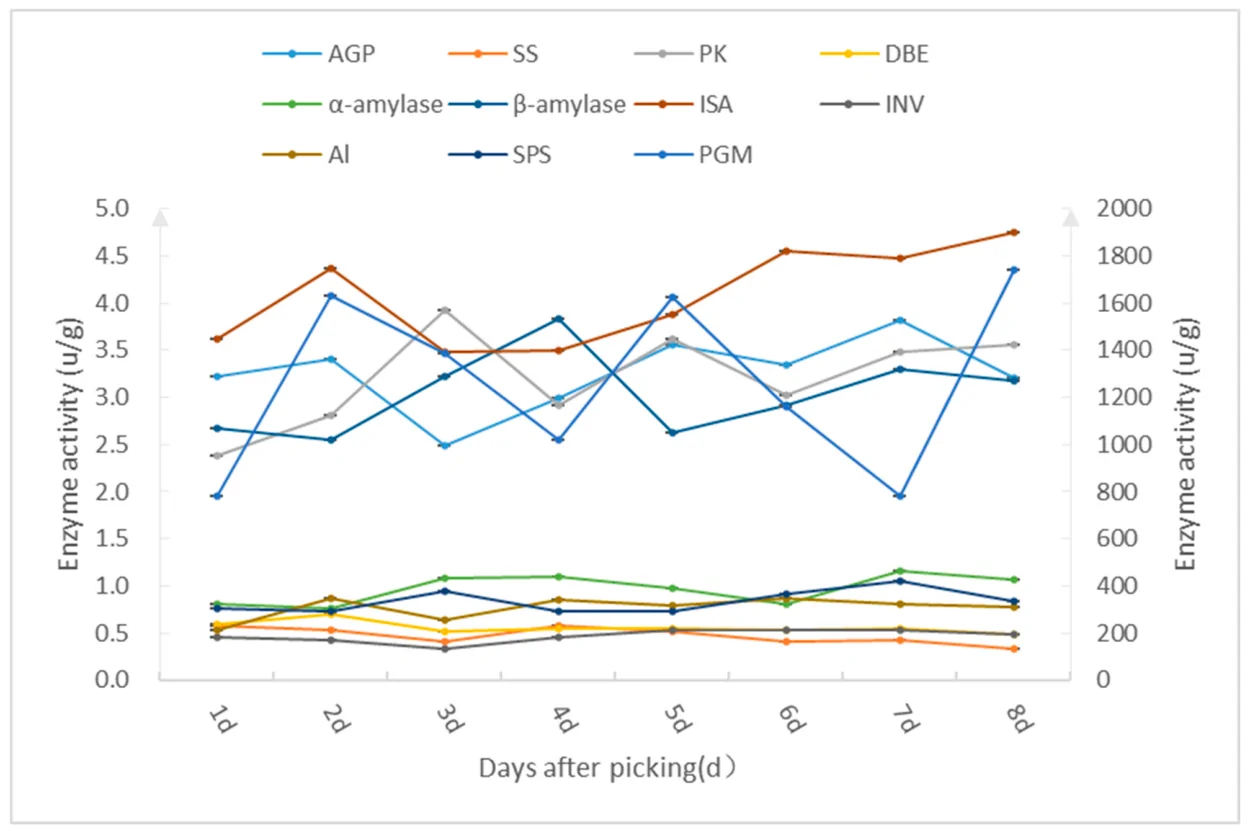
Dynamic changes in the activities of enzymes associated with sugar metabolism in ‘Renong No. 1’ fruits during ripening after picking. Note: AGP, adenosine diphosphate glucose pyrophosphorylase; SS, sucrose synthase; SPS, sucrose phosphate synthase; PK, protein kinase; DBE, starch debranching enzyme; PGM, phosphoglucomutase; ISA, isoamylase; INV, sucrose invertase; AI, acid invertase.

Dynamic changes in the activities of enzymes associated with sugar metabolism during the post-harvest ripening stage of ‘Renong No. 1’ mango fruits are shown in Figure 6. AI initially increased and then fluctuated slightly during the post-harvest ripening stage, while INV activity remained stable. AI activity was higher than that of INV during the whole post-harvest ripening. SS exhibited a gradual decreasing trend after picking and reached a minimum value at eight days. The trend exhibited by SPS activity significantly differed from that of SS activity, with SPS activity exhibiting an overall increasing trend. In addition, SPS activity was higher than that of SS during the entire fruit ripening stage after picking. AGP activity during postharvest ripening was similar on day 1 and day 8. PGM activity during the post-ripening stage was the highest and exhibited a fluctuating trend. PK activity initially increased and then fluctuated. ISA activity initially increased and subsequently decreased, then increased gradually to a maximum value. DBE activity initially increased and then decreased gradually.

### 2.3. Correlation Network Analysis of Total Soluble Solids, Soluble Sugar Content, Starch Content, Sweetness Values, and Activities of 11 Enzymes

#### 2.3.1. Mathematical Model Construction

**Figure 7:**
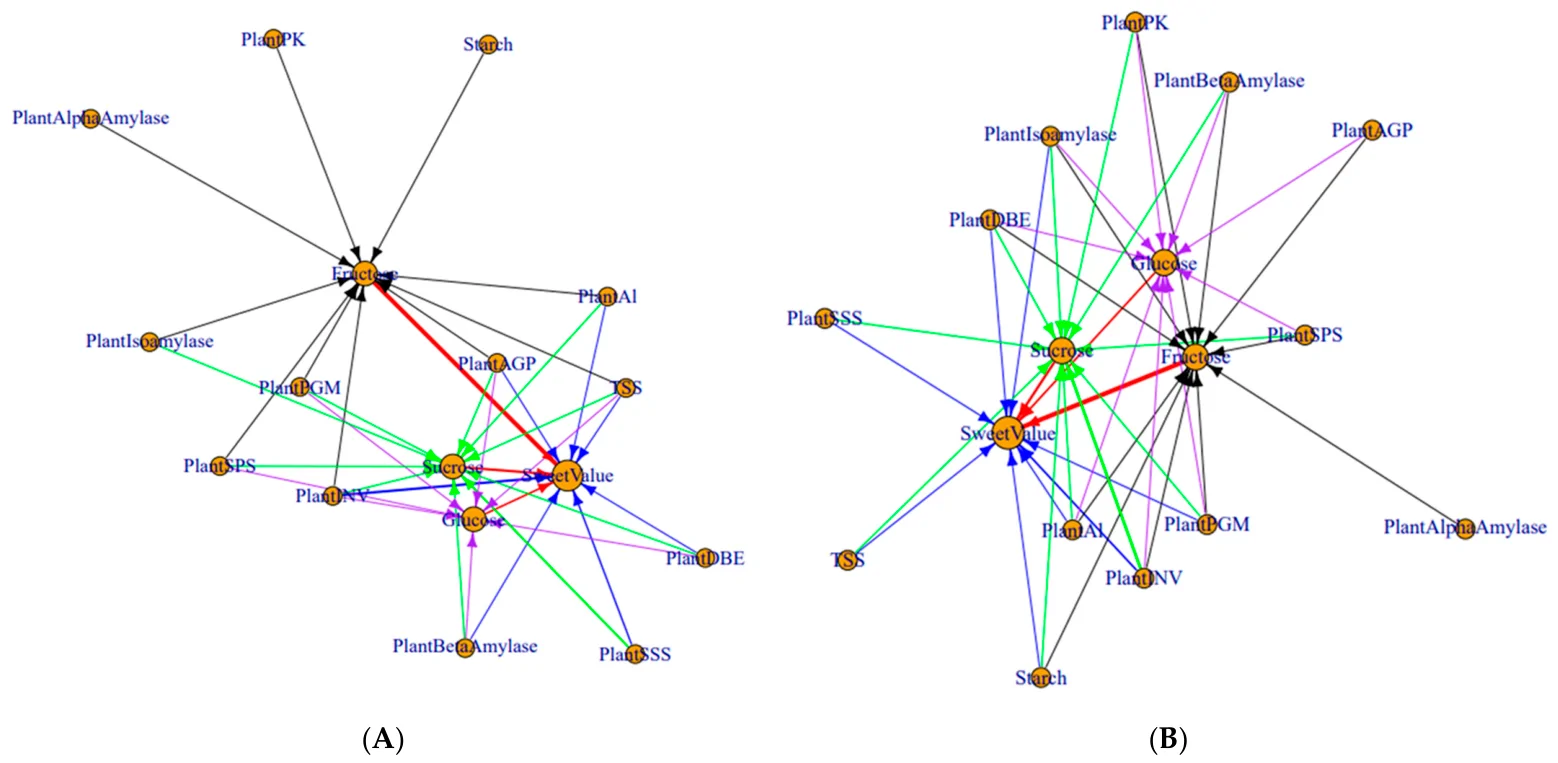
Correlation network of total soluble solids, starch content, soluble sugar content, and the activities of enzymes associated with sugar metabolism in ‘Renong No.1’ fruits. Note: (**A**) was from 50 to 140 days after flowering on trees (development to full ripening), and (**B**) was from 1 to 8 days postharvest. Greater line thickness corresponds to a stronger correlation between the two variables.

In order to verify the more precise mechanism of mango sugar metabolism, data (13 factors, including TSS, starch, AGP, SS, PK, DBE, PGM, α-amylase, β-amylase, ISA, INV, AI, and SPS on sweetness value, fructose, glucose, and sucrose) from tripartite materials of fruit development, on-tree ripening and postharvest ripening were analyzed respectively or collectively. The results were concretely visualized in detail by the corresponding network graphics (Figure 7), and the multiple regression equations were as follows.

Sweet Value/Sucrose/Fructose/Glucose = b0 + b1 × TSS + b2 × Starch + b3 × AGP + b4 × SS + b5 × PK + b6 × DBE + b7 × PGM + b8 × α-Amylase + b9 × β-Amylase + b10 × ISA + b11 × INV + b12 × AI + b13 × SPS.(1)

The coefficient bi (i = 1, 2, …, 13) represents the degree of influence of the ith factor on the sweetness value. Similarly, the sweetness value in Equation (1) was also substituted with fructose, glucose, and sucrose to determine the effect of the 13 factors on the three sugars.

Corresponding to Figure 7A (the statistical analysis was from 50 to 140 days after flowering, i.e., fruit on-tree development to full ripening), the equations were as follows:

Sweet Value = b0 + 507.6104 × TSS + 0.0000 × Starch + 1220.8488 × AGP + 2782.1551 × SS + 0.0000 × PK + 2115.9628 × DBE + 0.0000 × PGM + 0.0000 × α-Amylase + 172.1447 × β-Amylase + 0.0000 × ISA + 4283.6363 × INV − 1239.2313 × Al + 0.0000 × SPS(2)

Sucrose = b0 + 324.7599 × TSS + 0.0000 × Starch + 656.7960 × AGP + 3103.9855 × SS + 0.0000 × PK + 1412.8104 × DBE − 0.6155 × PGM + 0.0000 × α-Amylase + 218.7385 × β-Amylase − 124.0900 × ISA + 959.8746 × INV − 2192.7870 × Al + 1358.5346 × SPS(3)

Fructose = b0 + 83.3394 × TSS + 3.3733 × Starch + 305.6747 × AGP + 0.0000 × SS − 83.7792 × PK + 0.0000 × DBE + 0.0895 × PGM + 368.0104 × α-Amylase + 0.0000 × β-Amylase + 111.8021 × ISA + 1402.7959 × INV + 338.5056 × Al − 698.4897 × SPS(4)

Glucose = b0 + 56.3816 × TSS + 0.0000 × Starch + 176.2592 × AGP + 0.0000 × SS + 0.0000 × PK + 228.1749 × DBE + 0.1610 × PGM + 0.0000 × α-Amylase − 63.6643 × β-Amylase + 0.0000 × ISA + 1036.2415 × INV + 0.0000 × Al − 667.1473 × SPS(5)

Corresponding to Figure 7B (the statistical analysis was from postharvest day 1 to day 8), the equations were as follows:

Sweet Value = b0 + 79.5837 × TSS − 50.7630 × Starch + 0.0000 × AGP − 7909.2749 × SS + 0.0000 × PK − 1330.4457 × DBE − 0.5623 × PGM + 0.0000 × α-Amylase + 0.0000 × β-Amylase − 689.8341 × ISA + 11638.4862 × INV + 5310.3720 × Al + 0.0000 × SPS(6)

Sucrose = b0 + 90.8960 × TSS − 46.6710 × Starch + 0.0000 × AGP − 6123.7212 × SS + 236.7311 × PK − 3610.4238 × DBE + 1.0342 × PGM + 0.0000 × α-Amylase − 275.1201 × β-Amylase − 299.7360 × ISA + 15924.1409 × INV + 3463.1339 × Al − 462.3251 × SPS(7)

Fructose = b0 + 0.0000 × TSS + 4.7169 × Starch − 170.3737 × AGP + 0.0000 × SS − 80.2230 × PK + 737.7267 × DBE − 0.5769 × PGM − 138.9910 × α-Amylase + 159.8860 × β-Amylase − 104.4827 × ISA − 761.9089 × INV + 773.9589 × Al + 493.0499 × SPS(8)

Glucose = b0 + 0.0000 × TSS + 0.0000 × Starch − 97.0617 × AGP + 0.0000 × SS − 77.5436 × PK + 581.3381 × DBE − 0.5842 × PGM + 0.0000 × α-Amylase + 59.0282 × β-Amylase − 47.5125 × ISA − 1781.7872 × INV + 505.5739 × Al + 345.9200 × SPS(9)

Figure 7A,B uniformly show the effects of soluble sugars on sweetness value in the descending order of fructose > sucrose > glucose. The enzyme with the greatest effect on mango sweet value and soluble sugar was INV whether during the 50 to 140 days after flowering on-tree or 1 to 8 days postharvest. Equations (2)–(5) generally exhibited that starch has no positive effect on sweetness value or the contents of sucrose and glucose during on-tree fruit development and ripening. However, starch acts as an important negative influencing factor for sweetness value and the contents of sucrose and glucose during the postharvest ripening process (Equations (6)–(9)). This reveals that during fruit development, mangoes need to consume soluble sugars to accumulate large amounts of starch to support fruit volume enlargement, while after harvest, starch is strongly degraded to increase the content of soluble sugars. Besides, it is noteworthy that the effect of SS on carbohydrate regulation was bidirectional. SS strongly inhibited sucrose synthesis for collective driving force for starch accumulation in the mango fruit expansion stage, while it promoted sucrose synthesis markedly for sweet value improvement in the mango postharvest stage. Glucose content and fructose content were little affected by SS during the whole fruit development and ripening period (Equations (4), (5), (8) and (9), Table 4).

#### 2.3.2. Correlation Analysis Among Total Soluble Solids, Soluble Sugar Content, Starch Content, Sweetness Values, and Activities of Enzymes Associated with Sugar Metabolism

According to the results, fructose content was significantly positively correlated with AGP, α-amylase, AI, β-amylase, and SPS activities. Glucose content was significantly positively correlated with AGP activity. Sucrose content was significantly positively correlated with AGP, AI, PK, α-amylase, ISA, INV, and SPS activities. A significant negative correlation was observed between sucrose content and SS activity. Sweetness value was significantly positively correlated with AGP, α-amylase, AI, SPS, SS, and β-amylase activities (Table 5).

## 3. Discussion

### 3.1. Dynamic Changes in Total Soluble Solid, Soluble Sugar, and Starch Contents During the Growth and Post-Ripening Stages of Mango Fruits

During the fruit development stage, the dynamic changes in the soluble sugar content of ‘Renong No. 1’ are similar to those observed in ‘Tainong No. 1’ [19] and ‘KRS’ [30] mango fruits: the glucose, fructose, and sucrose contents all increased to varying degrees. The starch content of ‘Renong No. 1’ mango fruits increased slightly and continuously during the early growth stage, then decreased from the peak value but remained at a high level through the fruit growth and maturity stages. This trend is consistent with observations of starch content in ‘Tainong No. 1’ fruits [19], although the decline in ‘Renong No. 1’ is not as marked as that in ‘Tainong No. 1’. It is worth noting that sucrose rises from 21.95 mg/100 g FW (110 d) to 289.48 mg/100 g FW (125 d), a ~13-fold change in 15 days, and then to 1567.68 mg/100 g FW at 130 d (Table 1). This sharp transition, which has also been observed in ‘Yuexi No. 1’ and ‘Irwin’ [28,31], indicates that the fruit is approaching the end of its growth and begins a physiological switch to maturity. Correspondingly, starch accumulation stops. However, as this is a typical sucrose-accumulating fruit tree and its fruit acts as a strong sink, sucrose is continuously transported from leaves to the fruit to support the improvement of fruit quality.

During the post-ripening stage (after picking), the dynamic changes in fructose, glucose, and sucrose contents of ‘Renong No. 1’ mango fruits are almost similar to those of ‘Yuexi No. 1’ and ‘Aiwen’ mango fruits, but a great change was observed in starch content at day 8. That is, the starch content of ‘Renong No. 1’ mango fruits decreased by 43% (from 36.98 mg/g FW at 130 days after flowering on-tree to 21.24 mg/g FW at 8 days postharvest, Table 1 and Table 3). This reduction was smaller than the >90% starch decrease observed in both ‘Yuexi No. 1’ and ‘Irwin’ reported by Wang et al. [28,31]. This may be attributable to differences in measurement timing; this study only measured up to the eighth day post-harvest, at which point the fruits were still edible and their quality remained intact, whereas Wang’s measurement period was extended beyond the 10th day.

In this study, significant differences were observed in total soluble solid and soluble sugar contents between on-tree ripened fruits and postharvest fruits. Total soluble solid and soluble sugar contents exhibited a downward trend during the on-tree ripening stage, while they continually increased within the postharvest period, phenomena that are consistent with those of citrus [32], banana [33] and kiwi [34]. This is because during the on-tree ripening and senescence of fruits, fruits no longer function as the main sink organ. As ripening progresses, polysaccharides are degraded into soluble sugars, which then gradually diffuse out of the fruit and flow back to the parent tree. In contrast, during postharvest ripening, after polysaccharides are degraded, the resulting soluble sugars cannot be transported out of the detached fruit and instead accumulate within it.

### 3.2. Effects of Starch and Soluble Sugar Content on Sweetness Value of Mango Fruits

Mango is a starch-accumulating fruit and the fruit growth stage is dominated by starch accumulation. Starch is typically degraded to glucose by amylase when a fruit matures. Glucose is then converted to fructose, a reaction catalyzed by glucose-6-phosphate isomerase. The glucose and fructose generated are synthesized to sucrose by SPS and sucrose is further hydrolyzed to glucose and fructose by INV [5]. Therefore, various sugar contents in mango fruits are in different dynamic equilibriums at different stages of fruit growth or maturity.

The results of this study revealed that starch content was significantly positively correlated with glucose (r = 0.284) and fructose (r = 0.365); moreover, strong correlations were observed between sweetness value and total soluble sugar (r = 0.997) or individual sugars (Table 5). Unexpectedly, the correlation between starch content and sweetness value (r = 0.133) or total soluble sugar (r = 0.112) in ‘Renong No. 1’ mango fruits was weak (Table 5), which is inconsistent with the observations made for kiwifruit [35]. One explanation is that in this study sweetness value is a fixed linear combination of fructose, glucose, and sucrose; its near-perfect correlation with total soluble sugar and its strong correlations with the individual sugars are partly a mathematical consequence of this derivation rather than an independent physiological finding. Another explanation lies in the different carbohydrate storage strategies between mango (a sucrose-accumulating type) and kiwifruit (a hexose-accumulating type).

### 3.3. Correlations Among Soluble Sugar Content, Starch Content, and Activities of Enzymes Associated with Sugar Metabolism in Mango Fruits

Over the last 10 years, studies on enzymes associated with glucose metabolism in apple [4] and litchi [36] fruits have shown that the rate of glucose metabolism in fruits is influenced by the activities of key enzymes involved in glucose metabolism. The results of this study provided relevant experimental evidence.

Based on the results of the correlation analysis, the activities of PGM and DBE were not significantly correlated with the contents of sucrose, fructose, glucose, or starch, while the activities of AGP, AI, INV, PK, α-amylase, β-amylase, ISA, SS, and SPS were significantly correlated with glucose metabolism to varying degrees. The AGP activity of ‘Renong No. 1’ mango fruits positively correlated with starch content, which is consistent with the results of high starch accumulation in kiwifruits [35]. Qin [12] reported that PGM can be categorized into cytosolic PGM (cPGM) and plastid PGM (pPGM), and these promote sucrose accumulation and starch accumulation, respectively. Therefore, starch and soluble sugar contents of ‘Renong No. 1’ mango fruits could have been affected by the two types of PGM, although PGM had no significant correlation with soluble sugar and starch contents in this study. A previous study revealed that SS activity in mature mango fruits was significantly positively correlated with sucrose content [28], whereas SS activity was significantly negatively correlated with sucrose content in this study. The contrasting results could be attributed to variations in the maturity stages of the selected fruits and the different active members of SS subtypes. SS activity in ‘Renong No. 1’ mango fruits is associated with SS subtype I of sucrose degradation [37]. In this study, SPS activity was significantly positively correlated with sucrose and fructose contents, which is consistent with the findings of Wei et al. [28]. Sucrose invertase in mango fruits can be categorized into NI and AI [38]. The AI activity in the present study was higher than that of INV during the growth stage of ‘Renong No. 1’ mango fruits, which is consistent with the results of Li et al. [19]. Those results suggest that AI is the main type of invertase regulating sugar metabolism in ‘Renong No. 1’ mango fruits. RK1 protein kinase is a key factor that influences sugar signaling pathways by controlling key metabolic enzymes [39]. For example, antisense expression of SnRK1 homologous gene (*PKIN1)* in potatoes considerably decreases the expression of SS genes [40], resulting in low sucrose accumulation. In this study, PK activity in ‘Renong No. 1’ mango fruits was significantly positively correlated with sucrose content, whereas SS was significantly negatively correlated with sucrose content, which implies that PK did not induce sucrose accumulation through SS, but rather induced sucrose accumulation through SPS [41].

According to Ball [42], the function of DBE is to remove irregular branched sugar chains and inhibit the formation of glycogen so as to form a compact structure. DBE can be classified into ISA and branched amylase based on the substrate being hydrolyzed. Zeeman [43] observed that the starch content of leaf chloroplasts of *Arabidopsis* mutants lacking ISA was lower than that of control leaves, which explains the positive correlation observed between ISA and starch content in this study. According to Hussain [44], the ISA isoforms Stisa1 and Stisa3 exhibit starch hydrolase activity, which could explain the significant positive correlation observed between ISA and sucrose content in this study.

The activities of α-amylase, β-amylase, ISA, and DBE were not significantly correlated with starch content. This may be because the biochemical reaction of converting starch into soluble sugar in ‘Renong No. 1’ mango fruits during the mature stage is regulated not by a single amylase, but by multiple types of amylases, or by other molecules such as low-molecular-weight ferulic oligosaccharides [45].

### 3.4. Limitations of the Present Study

First, since all research in this study was conducted on only a single mango cultivar, the homogeneity of the nine selected trees was judged only through visual observation and prior experience, and all experiments were carried out at specific growth stages and under fixed cultivation conditions, the drawn conclusions have limited generalizability and require further validation. Second, this study only explores the regulatory mechanisms of carbohydrate metabolism from the perspective of soluble sugar composition and enzyme activity and does not delve deeply into the dynamic changes in key functional genes. Third, although the current sampling time points and measurement indicators are representative, dynamic monitoring with higher temporal resolution would enable more precise identification of key metabolic transition nodes and their corresponding regulatory timelines.

## 4. Materials and Methods

### 4.1. Plant Materials and Sampling

The fruits of the mango cultivar ‘Renong No. 1’ were from a mango orchard at the South Subtropical Crop Research Institute of the Chinese Academy of Tropical Agricultural Sciences, located in Zhanjiang, Guangdong, China (20°18′ N, 101°18′ E). The soil in the orchard is red-yellow soil. The orchard generally has natural conditions of 20–35 °C in temperature and 75–100% in relative humidity, and the entire orchard is subject to unified technical management covering irrigation, fertilization and pest control. ‘Renong No. 1’ is a conventional excellent high-yielding cultivar independently bred by the mango research group at the institute and approved by the National Tropical Crop Variety Approval Committee of China. The experimental trees of ‘Renong No. 1’ were 5 years old and had been grafted on 17-year old ‘Yuexi No. 1’ plants. Nine trees with consistent growth and inflorescence, and normal fruit growth and development, were selected for fruit sampling. Each experimental plot consisted of three trees, giving a total of three biological replicates.

**Figure 8:**
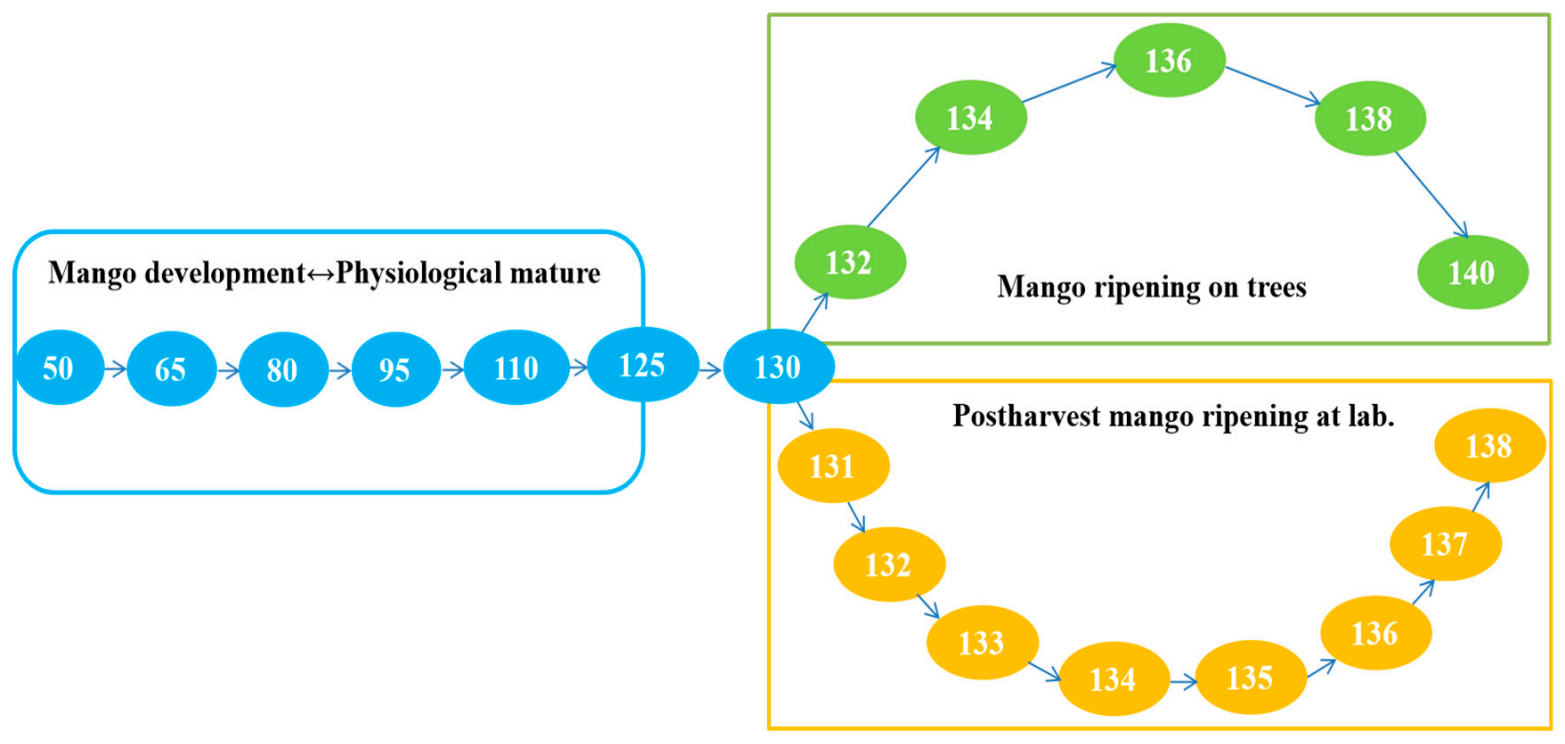
Sampling design.

**Figure 9:**
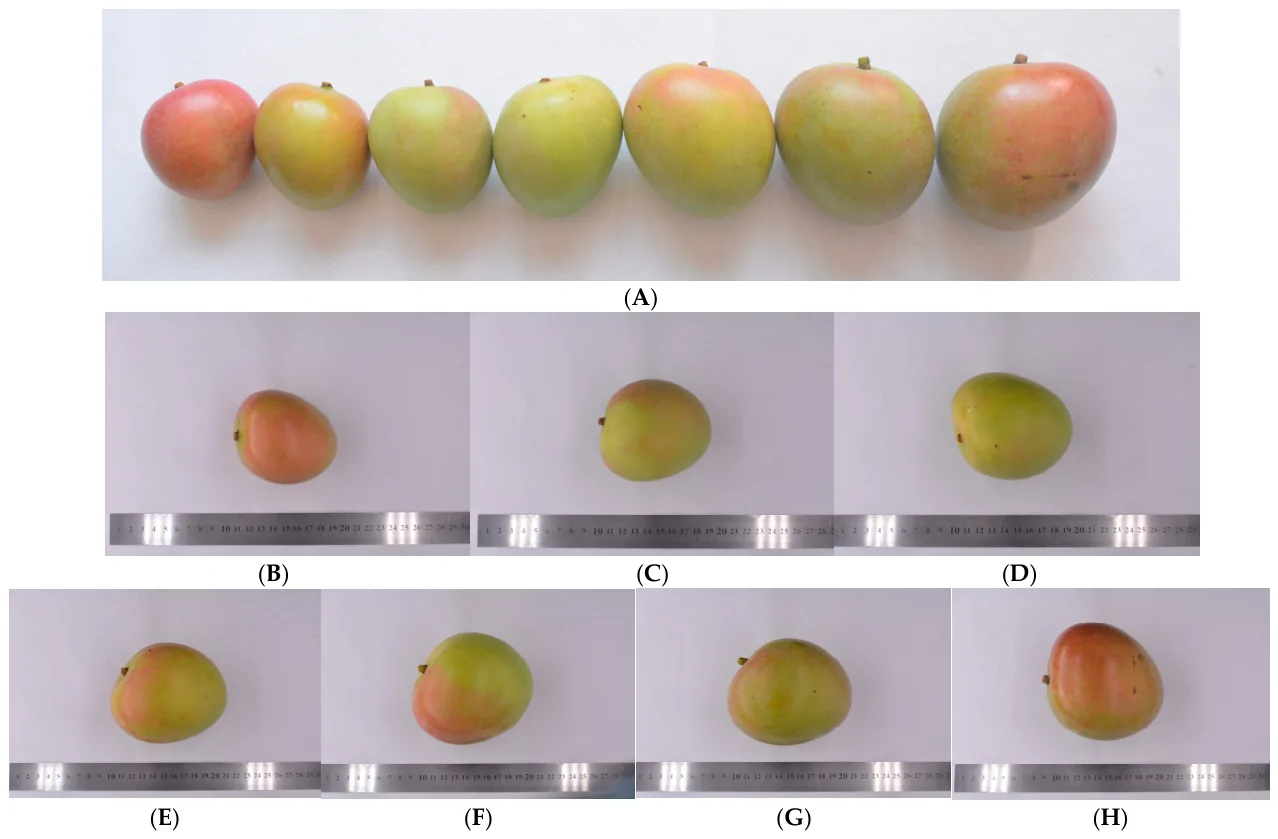
‘Renong No. 1’ mango fruits. Note: (**A**), general pattern; (**B**–**H**), from growth to physiological maturity stage.

The fruit sampling design is illustrated in Figure 8. Samples were first collected at 50 days after flowering and were then collected every 15 days until 130 days, when the fruits reached the maturity stage suitable for picking (Figure 9). During the post-ripening stage, fruit samples were divided into two groups: the on-tree ripening group and the off-tree ripening group. Based on the different rates of physiological change, fruits in the on-tree ripening group were first sampled at 130 days after flowering and then sampled every two days until 140 days when the on-tree fruits reached full ripeness. Fruits in the off-tree ripening group were picked at 130 days after flowering and stored in the laboratory (25–30 °C with 85–90% relative humidity) until they reached full ripeness at 138 days, and samples were collected daily during this period. Based on the above experimental design, data analysis was divided into three parts:

- 50–130 days after flowering (on-tree development to physiological maturity);
- 130–140 days after flowering (on-tree ripening);
- 1–8 days postharvest at lab (off-tree ripening).

Six fruits were included in each sample. Fruits with regular shape, uniform size and consistent maturity, and free from diseases and pests, were picked from the middle canopy of a sample tree. For each on-tree fruit sample, two fruits were picked from different directions of a single tree, and a total of six fruits were collected from the three trees in one plot. For the off-tree ripening group, uniform fruits were picked from the three trees in one plot and then transported to the lab for postharvest ripening, from which six fruits were randomly selected as a sample every day. For each sample, all six fruits were peeled, and flesh from the middle portion was collected, cut into 0.5–1 cm cubes, then mixed to prepare the test sample.

The total soluble solid (TSS) content of the fruit pulp was determined using a portion of the samples. The remaining samples were immediately frozen in liquid nitrogen, ground to powder, and stored in a refrigerator at −80 °C for subsequent analysis of starch and sugar contents and enzyme activity.

### 4.2. Determination of Total Soluble Solid Content

A portion of the mango pulp was chopped into pieces and mixed thoroughly. Juice was manually extracted from the pulp by wrapping the mixture with cotton clothes and squeezing. A digital hand-held refractometer (PAL-1; Atago Co., Ltd., Tokyo, Japan) was used to determine the TSS.

### 4.3. Determination of Soluble Sugar Content

The soluble sugar content was determined using HPLC according to the method described by Ma et al. [14]. Fruit pulp (1 g) was extracted with 85% ethanol and centrifuged. The supernatant was placed in a water bath maintained at 85 °C for complete evaporation of ethanol and then dissolved in 4 mL of water. The fruit pulp samples (1 mL) were filtered through 0.4 μm membrane filters and then analyzed by HPLC. A mixed standard stock solution (20 mg/mL) was prepared by accurately weighing glucose, fructose, and sucrose standards and dissolving them in ultrapure water. A series of mixed working standards at concentrations of 10, 5, 1.25, and 0.625 mg/mL were then obtained by transferring 50, 25, 6.25, and 3.125 mL aliquots of the stock solution into separate 100 mL volumetric flasks and diluting to the mark with ultrapure water. The resulting working solutions were employed for calibration curve generation.

**Figure 10:**
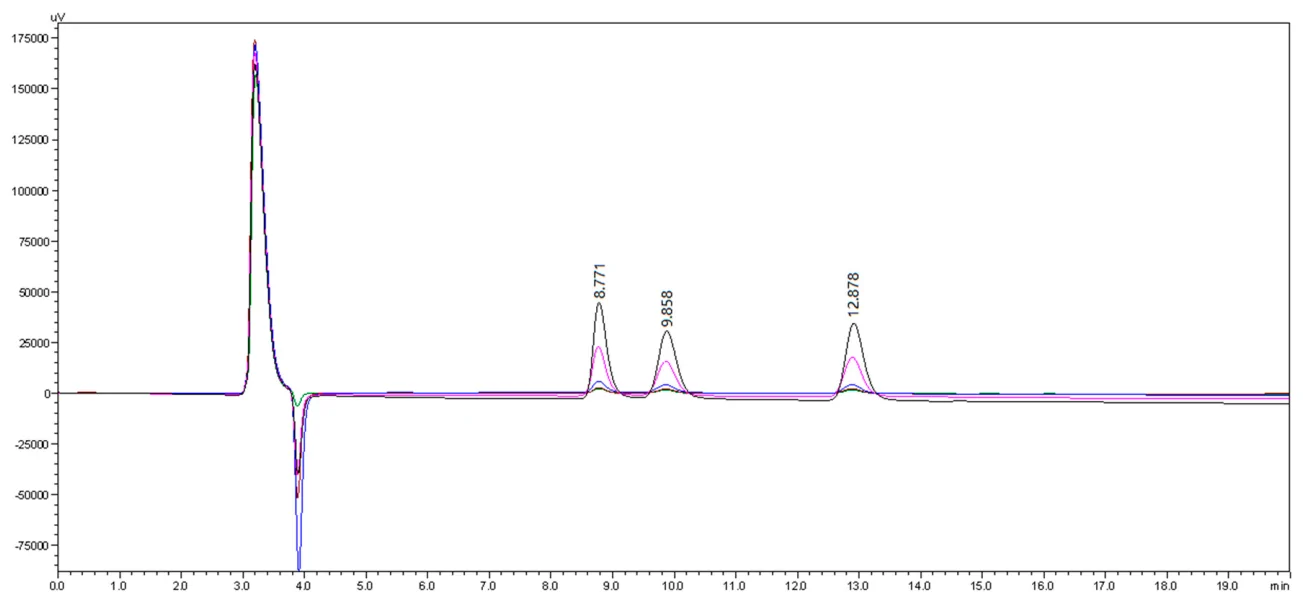
HPLC chromatogram of fructose, glucose and sucrose (superposition of standard samples).

**Figure 11:**
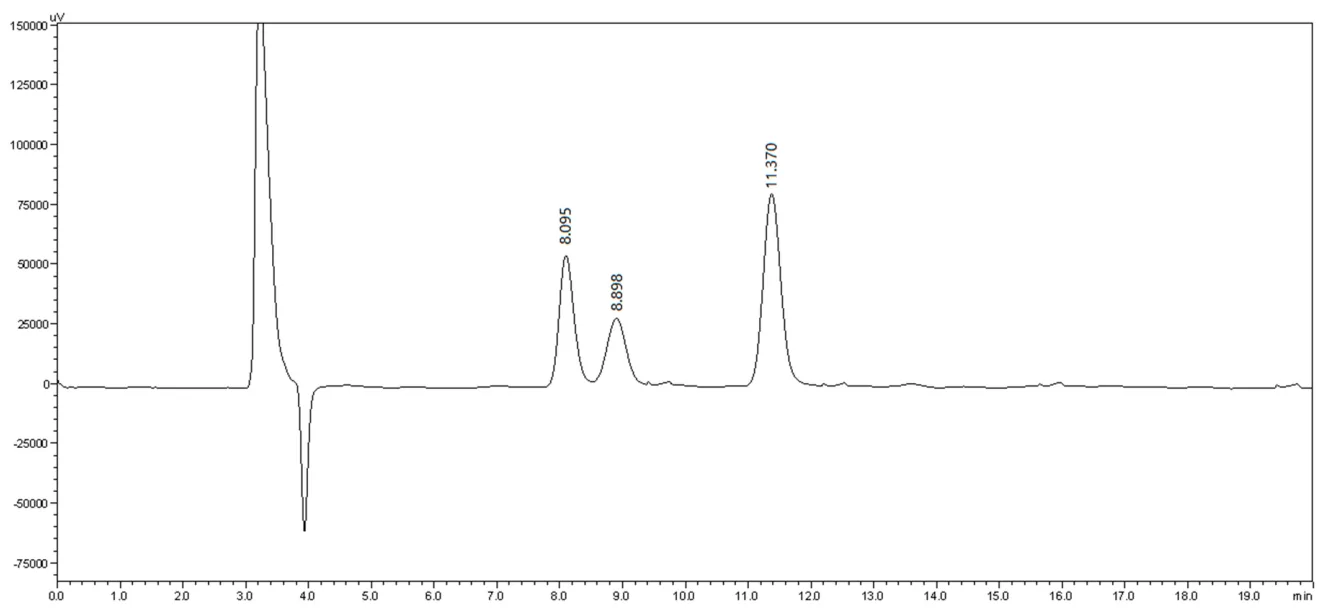
Representative HPLC chromatogram of fructose, glucose, and sucrose. Note: This example uses a real mango fruit sample collected at 8 days postharvest.

Chromatographic analysis was performed on a Waters e2695 high-performance liquid chromatography (HPLC) system equipped with a Waters 2414 refractive index detector (RID) and amino chromatographic column. Chromatographic conditions were set as follows: mobile phase consisted of acetonitrile and water (72:28, *v*/*v*) (used after ultrasonic degassing), flow rate was 1.0 mL/min, column and detector temperature maintained at 40 °C, injection volume was 10 μL, and the run time was 15 min. The soluble sugar content of each sample was calculated based on the peak area and standard curve. HPLC chromatograms are shown in Figure 10 and Figure 11.

### 4.4. Determination of Total Soluble Sugar Content and Sweetness Value

The total soluble sugar content was calculated as the sum of sucrose, glucose, and fructose.

Different types of sugars elicit distinct sweetness perceptions in humans, and these variations can be quantitatively characterized using the indicator of relative sweetness. With the sweetness intensity of sucrose set as the benchmark, and its relative sweetness defined as 1.00, the relative sweetness of fructose and glucose is determined as 1.75 and 0.75, respectively [46]. Therefore, the sweetness value = fructose content × 1.75 + sucrose content × 1.00 + glucose content × 0.75.

### 4.5. Determination of Starch Content

The soluble sugar and starch in the sample can be separated by using 80% ethanol. The acid hydrolysis method is further used to decompose the starch into glucose. The anthrone colorimetric method is used to measure the glucose content, and the starch content can be calculated. Based on the above principle, the plant starch content test kit (No: DF-2-Y) was used to determine starch content, according to the manufacturer’s instructions (Keming Biotechnology, Suzhou, China. https://www.cominbio.com).

### 4.6. Determination of Enzyme Activity

The activities of enzymes associated with sugar metabolism, including AGP, SS, PK, PGM, α-amylase, β-amylase, DBE, ISA, INV, AI, and SPS, were determined using assay kits (Shanghai Enzyme-linked Biotechnology, Shanghai, China, Table 6). The experimental procedures were performed according to the manufacturer’s instructions. Sample pre-treatment was performed as follows: mango pulp samples from the same replicate group were weighed to 50 mg, then 0.45 mL of 0.01 mol/L PBS (Phosphate Buffered Saline) buffer (pH 7.4) was added, and the specimen was homogenized with a homogenizer. The homogenized mixture was centrifuged at 2000–3000 rpm for 20 min, after which the supernatant was carefully collected and aliquoted. One aliquot was used for immediate testing, and the remaining aliquots were stored at −80 °C for later use. Detection was performed with a Rayto RT-6100 microplate reader (Rayto Life and Analytical Sciences Co., Ltd., Shenzhen, China) at a detection wavelength of 450 nm.

### 4.7. Data Analysis

Data obtained by HPLC were analyzed using Analyst 1.5.2 (AB SCIEX, Foster City, CA, USA). Statistical data, including one-way ANOVA with Duncan’s multiple range test, were analyzed using MS Excel 2010 (Microsoft Corp., Redmond, WA, USA) and SPSS 17.0 (SPSS Inc., Chicago, IL, USA). R 3.6.1 was used for data conversion and network construction, with the “lars 1.3” and “igraph 1.6.0” packages used for LASSO (Least Absolute Shrinkage and Selection Operator) regression analysis and plotting, respectively.

## 5. Conclusions

This study systematically characterized the variation trends of mango carbohydrate metabolism throughout the fruit development process. Glucose, fructose, sucrose and starch, as well as the activities of 11 enzymes associated with sugar metabolism, were tested. Simultaneously, systematic and in-depth analyses were made for their trends in the measured values. Furthermore, new mathematical models with quantitative equations and the networks of soluble sugars and the related enzymes with visualized graphs were adopted to represent the relationships among all the indicators. As a result, the quantitative equations and the intuitive visualizations from the different data groupings respectively revealed the genuine functional dependencies and the correlation intensities among the indicators under different grouping conditions. The findings of this study can be used to advance mango cultivation and breeding. For instance, the variation patterns of soluble sugar content in mangoes allows us to develop criteria for determining physiological maturity stage and optimal harvest time. Additionally, the key enzymes affecting sucrose metabolism in mango can be used to select breeding targets.

## Figures and Tables

**Table 1:** Changes in soluble sugars, starch, total soluble solid contents, and sweetness value of fruits during growth and the physiological maturity stage.

Time	Fructose (mg/100 g FW)	Glucose (mg/100 g FW)	Sucrose (mg/100 g FW)	Total Soluble Solid (%)	Total Soluble Sugar (mg/100 g FW)	Sweetness Value ^1^ (Index)	Starch (mg/g FW)
50 d	979.07 ± 97.93 ^(cd)^	762.68 ± 40.76 ^(bcd)^	22.08 ± 0.04 ^(c)^	8.20 ± 1.36 ^(b)^	1763.84 ± 161.40 ^(d)^	2307.47 ± 239.79 ^(cd)^	27.99 ± 1.31 ^(c)^
65 d	760.31 ± 206.27 ^(d)^	732.27 ± 91.26 ^(cd)^	22.03 ± 0.15 ^(c)^	7.62 ± 0.41 ^(c)^	1514.61 ± 356.29 ^(d)^	1901.78 ± 518.59 ^(d)^	29.07 ± 0.95 ^(c)^
80 d	1149.52 ± 92.30 ^(c)^	867.59 ± 35.89 ^(bc)^	22.08 ± 0.15 ^(c)^	7.52 ± 0.48 ^(c)^	2039.19 ± 79.84 ^(cd)^	2684.44 ± 171.05 ^(c)^	47.14 ± 2.57 ^(a)^
95 d	1158.48 ± 56.91 ^(c)^	659.87 ± 59.59 ^(d)^	21.88 ± 0.02 ^(c)^	5.66 ± 0.23 ^(d)^	1840.23 ± 141.51 ^(d)^	2544.13 ± 175.47 ^(cd)^	38.81 ± 2.03 ^(b)^
110 d	1589.42 ± 43.16 ^(b)^	909.76 ± 43.83 ^(b)^	21.95 ± 0.09 ^(c)^	8.5 ± 0.57 ^(b)^	2521.14 ± 92.39 ^(bc)^	3485.76 ± 118.01 ^(b)^	27.04 ± 4.42 ^(c)^
125 d	1473.74 ± 72.75 ^(b)^	831.73 ± 12.94 ^(bc)^	289.48 ± 26.11 ^(bc)^	7.88 ± 0.62 ^(b)^	2594.95 ± 105.80 ^(b)^	3492.32 ± 176.16 ^(b)^	36.79 ± 3.63 ^(b)^
130 d	2219.15 ± 191.92 ^(a)^	1363.38 ± 118.72 ^(a)^	1567.68 ± 313.31 ^(a)^	11.7 ± 0.29 ^(a)^	5150.22 ± 614.55 ^(a)^	6473.74 ± 737.98 ^(a)^	36.98 ± 1.47 ^(b)^

Note: ^1^ Sweetness value is expressed as a relative index according to Yao’s formula where SV = fructose content × 1.75 + sucrose content × 1.00 + glucose content × 0.75. Numbers with the same letter in the same column are not significantly different at *p* < 0.05.

**Table 2:** Changes in soluble sugars, starch, total soluble solid contents, and sweetness value of mango fruits during the on-tree ripening stage.

Time	Fructose (mg/100 g FW)	Glucose (mg/100 g FW)	Sucrose (mg/100 g FW)	Total Soluble Solid (%)	Total Soluble Sugar (mg/100 g FW)	Sweetness Value (Index)	Starch (mg/g FW)
130 d	2219.15 ± 191.92 ^(a)^	1363.38 ± 118.72 ^(a)^	1567.68 ± 313.31 ^(b)^	11.70 ± 0.29 ^(a)^	5150.22 ± 614.55 ^(ab)^	6473.74 ± 737.98 ^(a)^	36.98 ± 1.47 ^(a)^
132 d	1351.78 ± 23.83 ^(c)^	619.11 ± 18.33 ^(c)^	1399.31 ± 163.32 ^(b)^	7.43 ± 1.80 ^(bc)^	2110.83 ± 42.05 ^(d)^	2969.89 ± 61.04 ^(b)^	34.56 ± 2.54 ^(a)^
134 d	1495.86 ± 58.93 ^(bc)^	825.24 ± 60.85 ^(bc)^	2943.91 ± 47.80 ^(a)^	8.46 ± 1.00 ^(b)^	5265.027 ± 193.26 ^(a)^	6180.61 ± 226.13 ^(a)^	34.47 ± 1.88 ^(a)^
136 d	2179.74 ± 304.71 ^(ab)^	1376.33 ± 199.30 ^(a)^	1565.53 ± 30.47 ^(b)^	10.93 ± 2.21 ^(ab)^	4121.60 ± 648.87 ^(c)^	5412.33 ± 867.75 ^(a)^	28.66 ± 2.75 ^(b)^
138 d	1903.17 ± 23.67 ^(bc)^	1088.37 ± 31.39 ^(b)^	1591.83 ± 19.69 ^(b)^	10.07 ± 2.52 ^(ab)^	4583.38 ± 66.09 ^(b)^	5738.67 ± 80.72 ^(a)^	33.49 ± 3.35 ^(a)^
140 d	1036.27 ± 44.69 ^(d)^	498.34 ± 42.87 ^(d)^	265.56 ± 6.53 ^(c)^	6.38 ± 0.83 ^(c)^	1800.191 ± 104.19 ^(d)^	2452.80 ± 131.08 ^(c)^	21.15 ± 5.55 ^(c)^

Note: Sweetness value is expressed as a relative index according to Yao’s formula where SV = fructose content × 1.75 + sucrose content × 1.00 + glucose content × 0.75. Numbers with the same letter in the same column are not significantly different at *p* < 0.05.

**Table 3:** Changes in soluble sugars, starch, total soluble solid contents, and sweetness value of fruits during postharvest ripening stage.

Time	Fructose (mg/100 g FW)	Glucose (mg/100 g FW)	Sucrose (mg/100 g FW)	Total Soluble Solid (%)	Total Soluble Sugar (mg/100 g FW)	Sweetness Value (Index)	Starch (mg/g FW)
1 d	2164.84 ± 102.82 ^(bc)^	1388.07 ± 105.05 ^(ab)^	435.43 ± 56.41 ^(f)^	11.16 ± 2.22 ^(a)^	3988.35 ± 268.44 ^(e)^	5264.97 ± 326.25 ^(d)^	40.23 ± 1.53 ^(a)^
2 d	2021.81 ± 24.09 ^(c)^	1227.35 ± 24.51 ^(b)^	1854.63 ± 107.43 ^(d)^	11.20 ± 2.47 ^(a)^	5103.81 ± 105.72 ^(d)^	6313.33 ± 96.64 ^(c)^	35.73 ± 1.85 ^(b)^
3 d	2227.70 ± 7.34 ^(bc)^	1452.62 ± 47.22 ^(a)^	1174.17 ± 209.82 ^(e)^	11.63 ± 1.81 ^(a)^	4854.50 ± 257.12 ^(d)^	6162.12 ± 248.03 ^(c)^	35.94 ± 3.64 ^(b)^
4 d	2457.36 ± 144.45 ^(a)^	1541.90 ± 103.05 ^(a)^	2427.42 ± 139.58 ^(c)^	12.43 ± 0.66 ^(a)^	6426.72 ± 456.51 ^(c)^	7884.29 ± 557.36 ^(b)^	32.16 ± 0.34 ^(c)^
5 d	1549.30 ± 78.04 ^(d)^	726.16 ± 39.35 ^(c)^	4995.03 ± 101.47 ^(a)^	14.20 ± 0.49 ^(a)^	7270.50 ± 267.51 ^(b)^	8250.93 ± 327.00 ^(b)^	32.09 ± 1.33 ^(c)^
6 d	2127.80 ± 58.53 ^(c)^	1257.83 ± 62.78 ^(b)^	4931.88 ± 192.29 ^(a)^	14.40 ± 0.61 ^(a)^	8317.52 ± 380.03 ^(a)^	9598.92 ± 412.87 ^(a)^	31.86 ± 3.17 ^(c)^
7 d	2370.37 ± 171.32 ^(ab)^	1464.92 ± 167.75 ^(a)^	3744.26 ± 135.45 ^(b)^	14.46 ± 0.49 ^(a)^	7579.57 ± 535.06 ^(b)^	8991.12 ± 635.37 ^(a)^	31.29 ± 1.79 ^(c)^
8 d	1529.34 ± 41.18 ^(d)^	596.47 ± 13.91 ^(c)^	4964.93 ± 235.73 ^(a)^	15.23 ± 0.66 ^(a)^	7090.75 ± 187.39 ^(b)^	8088.64 ± 166.32 ^(a)^	21.24 ± 1.81 ^(d)^

Note: Sweetness value is expressed as a relative index according to Yao’s formula where SV = fructose content × 1.75 + sucrose content × 1.00 + glucose content × 0.75. Numbers with the same letter in the same column are not significantly different at *p* < 0.05.

**Table 4:** Summary of effects on mango sweet value and soluble sugar contents from all the indexes.

Indexes		Figure 7A (50–140 Days On-Tree Development to Ripening)	Figure 7B (1–8 Days Postharvest)
Sweet value	The most influential	INV, −SS, −DBE	INV, SS,−AI, AGP
	The least significant	AGP, PK, α-Amylase, β-Amylase, SPS	Starch, PK, PGM, α-Amylase, ISA, SPS
	Negatively influential	−Starch, −PGM, −SS, −DBE	−AI
Sucrose	The most influential	INV, −SS, −DBE	SS, −AI, DBE, SPS
	The least significant	AGP, α-Amylase, β-Amylase	Starch, PK, α-Amylase
	Negatively influential	−SS, −DBE, −SPS, −ISA, −β-Amylase, −Starch	−AI, −ISA, −PGM
Glucose	The most influential	DBE, AI, SPS	INV, −SPS, DBE
	The least significant	TSS, Starch, SS, α-Amylase	Starch, SS, PK, α-Amylase, ISA, AI
	Negatively influential	−INV, −AGP, −PK, −ISA, −PGM	−SPS, −β-Amylase
Fructose	The most influential	AI, −INV, DBE, SPS	−SPS, α-Amylase, AGP
	The least significant	TSS, SS	SS, DBE, β-Amylase
	Negatively influential	−INV, −AGP, −α-Amylase, −ISA	−SPS, −PK

Note: − means negative impact of the enzymatic activity.

**Table 5:** Correlations among TSS, soluble sugar content, starch content, sweetness values, and activities of enzymes associated with sugar metabolism.

Index	Fructose	Glucose	Sucrose	Starch	AGPase	SS	PK	DBE	PGM	α-Amylase	β-Amylase	ISA	INV	AI	SPS	SV	TSS
Fructose	1	0.926 **	0.456 *	0.284	0.654 **	−0.184	0.225	−0.14	0.035	0.636 **	0.557 *	0.357	0.266	0.635 **	0.560 *	0.775 **	0.721 **
Glucose	—	1	0.261	0.365	0.465 *	−0.177	0.05	−0.164	−0.118	0.442	0.403	0.148	0.071	0.39	0.372	0.614 **	0.558 *
Sucrose	—	—	1	−0.004	0.733 **	−0.498 *	0.452 *	−0.342	0.142	0.482 *	0.328	0.483 *	0.451 *	0.654 **	0.506 *	0.915 **	0.944 **
Starch	—	—	—	1	0.387	0.224	−0.209	0.181	0.251	0.022	−0.105	0.258	−0.394	−0.04	−0.043	0.133	0.112
AGPase	—	—	—	—	1	−0.149	0.188	−0.125	0.222	0.436	0.258	0.610 **	0.382	0.708 **	0.542 *	0.811 **	0.802 **
SS	—	—	—	—	—	1	−0.371	0.583 **	0.064	−0.372	−0.149	−0.075	−0.151	−0.171	−0.352	−0.445 *	−0.467 *
PK	—	—	—	—	—	—	1	−0.133	0.169	0.669 **	0.42	0.102	0.104	0.319	0.451 *	0.414	0.422
DBE	—	—	—	—	—	—	—	1	0.35	−0.02	0.194	0.13	−0.287	−0.081	−0.365	−0.314	−0.329
PGM	—	—	—	—	—	—	—	—	1	0.272	0.106	0.465 *	−0.326	0.218	−0.061	0.104	0.105
α-amylase	—	—	—	—	—	—	—	—	—	1	0.793 **	0.307	0.132	0.483 *	0.505 *	0.619 **	0.593 **
β-amylase	—	—	—	—	—	—	—	—	—	—	1	0.199	0.21	0.487 *	0.411	0.475 *	0.446 *
ISA	—	—	—	—	—	—	—	—	—	—	—	1	0.252	0.646 **	0.553 *	0.492 *	0.489 *
INV	—	—	—	—	—	—	—	—	—	—	—	—	1	0.495 *	0.577 **	0.430	0.433
AI	—	—	—	—	—	—	—	—	—	—	—	—	—	1	0.578 **	0.739 **	0.724 **
SPS	—	—	—	—	—	—	—	—	—	—	—	—	—	—	1	0.603 **	0.585 **
SV	—	—	—	—	—	—	—	—	—	—	—	—	—	—	—	1	0.997 **
TSS	—	—	—	—	—	—	—	—	—	—	—	—	—	—	—	—	1

Note: − means negative correlation; ** indicates significant correlation (*p* < 0.01); * indicates appreciable correlation (*p* < 0.05); —, none. SV, sweetness value; TSS, total soluble sugar.

**Table 6:** Methods and coefficients of variation of enzyme-linked immunosorbent assays.

Commercial Assay Kit	Supplier	Item Number	Limit of Detection	Coefficient of Variation (CV)
Plant β-Amylase ELISA Kit	Shanghai Enzyme-linked Biotechnology Co., Ltd.	YJ207960	250~4000 U/L	Intra-assay CV: ≤10%; Inter-assay CV (between plates): ≤15%
Plant AGPase ELISA Kit		YJ503647	1~16 U/mL	
Plant Sucrose Synthase (SS) ELISA Kit		YJ281580	6~96 U/mL	
Plant Protein Kinase (PK) ELISA Kit		YJ281704	12.5~200 U/mL	
Plant Starch Debranching Enzyme (DBE) Kit		YJ203625	25~400 U/L	
Plant Phosphoglucomutase (PGM) ELISA Kit		YJ303570	30~480 U/mL	
Plant α-Amylase ELISA Kit		YJ303253	25~400 U/L	
Plant Isoamylase ELISA Kit		YJ303290	40~640 U/L	
Plant Invertase (INV) ELISA Kit		YJ988025	2~32 U/mL	
Plant Acid Invertase (AI) ELISA Kit		YJ520941	50~800 U/mL	
Plant Sucrose Phosphate Synthase (SPS) ELISA Kit		YJ016954	0.5~8 U/mL	

## Data Availability

The original contributions presented in this study are included in the article. Further inquiries can be directed to the corresponding authors.

## References

[1] FAO (2022). https://www.fao.org/faostat/zh/#data/QCL.

[2] Liu B.D., Xin Q., Zhang M., Chen J.H., Lu Q.C., Zhou X.Q., Li X.X., Zhang W.L., Feng W., Pei H.S. (2023). Research progress on mango post-harvest ripening physiology and the regulatory technologies. Foods.

[3] Huang G.D., Chen Y.S., Su M.H., Luo S.X., Li R.W., Wang Y.R. (2024). Protection, Utilization status quo and development path of mango germplasm resources in Guangxi. Agric. Res. Appl..

[4] Wang Y.Z., Zhang D.P. (2001). A Study on the relationships between acid invertase, sucrose synthase and sucrose metabolism in ‘Red Fuji’apple fruit. Acta Hortic. Sin..

[5] Zhang L., Chen K.S. (2006). Molecular Physiology of Fruit Quality Development and Regulation.

[6] Yonemori K., Hirano K., Sugiura A. (1995). Growth inhibition of persimmon fruit caused by calyx lobe removal and possible involvement of endogenous hormones. Sci. Hortic..

[7] Yamada M., Yamane H., Takano Y., Ukai Y. (1997). Estimation of the proportion of offspring having soluble solids content in fruit exceeding a given critical Value in Japanese persimmon. Euphytica.

[8] Feng B.S., Liu L.X., Sun J., Len P., Wang L., Guo Y.Y., Min D.D., Liu Y.G. (2024). Combined metabolome and transcriptome analyses of quality components and related molecular regulatory mechanisms during the ripening of Huangjin Peach. Sci. Hortic..

[9] Peng Z.Z., Fu D.Q. (2023). Effects of 1-methylcyclopropene treatment on the quality of red ‘Fuji’ apples fruit during short-term storage. Food Qual. Saf..

[10] Schaffer A.A., Miron D., Petreikov M., Fogelman M., Spiegelman M., Bnei-Moshe Z., Shen S., Granot D., Hadas R., Dai N. (1999). Modification of carbohydrate content in developing tomato fruit. HortScience.

[11] Coombe B.G. (1989). The grape berry as a sink. Acta Hortic..

[12] Qin Q.P., Zhang S.L., Xie M., Chen J.M. (2005). Progress on the research of the molecular regulation of sugar content and composition in fruit. J. Fruit Sci..

[13] Zhang A., Yang X., Liu D., Luo Z., Zhang Z., Huo J., Han D., Zhang Y., Zhang L. (2025). Genome-wide characterization of kiwifruit invertase gene family reveals roles of *AcCWINV4* in sugar accumulation and cold tolerance. Int. J. Mol. Sci..

[14] Chen C., Chen H., Yang W., Li J., Tang W., Gong R. (2022). Transcriptomic and metabolomic analysis of quality changes during sweet cherry fruit development and mining of related genes. Int. J. Mol. Sci..

[15] Gou N., Chen C., Huang M., Zhang Y., Bai H., Li H., Wang L., Wuyun T. (2023). Transcriptome and metabolome analyses reveal sugar and acid accumulation during apricot fruit development. Int. J. Mol. Sci..

[16] Sun H., Wei R., Yin K., Meng D., Wu S., Bai H., Li Z., Kashif M., Liang Z., Chen S. (2025). Discovery and interaction of four key biosynthetic enzymes under co-regulation for dopamine biosynthesis with marine *Meyerozyma guilliermondii* GXDK6 and *Bacillus aryabhattai* NM1-A2. Int. J. Biol. Macromol..

[17] Bernardes-silva A.P., Nascimento J.R., Lajolo F.M., Cordenunsi B.R. (2008). Starch mobilization and sucrose accumulation in the pulp of Keitt mangoes during postharvest ripening. J. Food Biochem..

[18] Maldonado-Celis M.E., Yahia E.M., Bedoya R., Landázuri P., Loango N., Aguillón J., Restrepo B., Guerrero Ospina J.C. (2019). Chemical composition of mango (*Mangifera indica* L.) fruit: Nutritional and phytochemical compounds. Front. Plant Sci..

[19] Li L., Wu H.X., Ma X.W., Xu W.T., Liang Q.Z., Zhan R.L., Wang S.B. (2020). Transcriptional mechanism of differential sugar accumulation in pulp of two contrasting mango (*Mangifera indica* L.) cultivars. Genomics.

[20] Seymour G.B., Taylor J.E., Tucker G.A. (1996). Biochemistry of Fruit Ripening.

[21] Medlicott A.P., Thompson A.K. (1985). Analysis of sugars and organic acids in ripening mango fruits (*Mangifera indica* L. var Keitt) by high performance liquid chromatography. J. Sci. Food Agric..

[22] Simão R., Silva A.P.F.B., Gonc F.H., Nascimento J.R.D., Louro R., Lajolo F., Cordenunsi B. (2008). Mango starch degradation. I. a microscopic view of the granule during ripening. J. Agric. Food Chem..

[23] Sun J.D., Loboda T., Sung S.J.S., Black C.C. (1992). Sucrose synthase in wild tomato, *Lycopersicon chemieleleuskii*, and tomato fruit sink strength. Plant Physiol..

[24] Shah I.H., Wu J.H., Li X.Y., Hameed M.K., Manzoor M.A., Li P.L., Zhang Y.D., Niu Q.L., Chang L.Y. (2024). Exploring the role of nitrogen and potassium in photosynthesis implications for sugar: Accumulation and translocation in horticultural crops. Sci. Hortic..

[25] Lowell C.A., Tomlinson P.T., Koch K.E. (1989). Sucrose-metabolizing enzymes in transport tissue and adjacent sink structures in developing citrus fruit. Plant Physiol..

[26] Komatsu A., Moriguchi T., Koyama K., Omura M., Akihama T. (2002). Analysis of sucrose synthase genes in citrus suggests different roles and phylogenetic relationships. J. Exp. Bot..

[27] Mattoo A.K., Modi V.V. (1970). Biochemical aspects of ripening and chilling injury in mango fruit. Proceedings of the Tropical Product Institute Conference.

[28] Wei C.B., Wu H.X., Ma W.H., Wang S.B., Sun G.M. (2008). Sucrose metabolism in Irwin mango (*Mangifera indica* L.) during maturation. Southwest China J. Agric. Sci..

[29] Godoy A., Morita R.J., Cordenunsi B.R., Lajolo F., Nascimento J. (2009). Expression analysis of a set of genes related to the ripening of bananas and mangoes. Braz. J. Plant Physiol..

[30] Wu H.X., Yao Q.S., Wang S.B., Ma X.W., Zhan R.L. (2016). The relationship between sugar accumulation and related enzyme activities during the development process of ‘KRS’ mango fruits. J. Anhui Agric. Sci..

[31] Wei C.B., Wu H.X., Ma W.H., Wang S.B., Sun G.M. (2009). Sucrose metabolism in fruits of mango cultivar Yuexi No. 1 (*Mangifera indica* L.) at the mature stage. Chin. J. Trop. Crops.

[32] Ochiki S.N., Chen T.X., Meng Z.X., Zhou J.H., Gao Z.X., Deng Y., Luan M.B. (2024). Characterization of ATP-dependent phosphofructokinase genes during ripening and their modulation by phytohormones during postharvest storage of citrus fruits (*Citrus reticulata* Blanco.). Plant Physiol. Biochem..

[33] Wei W., Yang Y.Y., Wu C.J., Kuang J.F., Lu W.J., Chen J.Y., Shan W. (2023). *MaNAC19–MaXB3* regulatory module mediates sucrose synthesis in banana fruit during ripening. Int. J. Biol. Macromol..

[34] Wang P., Feng X., Jiang J., Yan P., Li Z., Luo W., Chen Y., Ye W. (2023). Transcriptome analysis reveals fruit quality formation in *Actinidia eriantha* Benth. Plants.

[35] Zhang H.Q., Xie M., Zhang C., Yang L.Q., Zhang Z., Xiao J.P., Zhou L.Q. (2014). Difference in starch accumulation and characterization of sugar metabolism during fruit development of kiwi fruit. Sci. Agric. Sin..

[36] Wang H.C., Huang H.B., Huang X.M. (2003). Sugar accumulation and related enzyme activities in the litchi fruit of ‘Nuomici’ and ‘Feizixiao’. Acta Hortic. Sin..

[37] Tanase K., Yamaki S. (2000). Purification and characterization of two sucrose synthase isoforms from Japanese pear fruit. Plant Cell Physiol..

[38] Wan H., Wu L., Yang Y., Zhou G., Ruan Y. (2018). Evolution of sucrose metabolism: The dichotomy of invertases and beyond. Trends Plant Sci..

[39] Sugden C., Donaghy P.G., Halford N.G., Hardie D.G. (1999). Two SNF1-related protein kinases from spinach leaf phosphorylate and inactivate 3-hydroxy-3-methylglutaryl-coenzyme a reductase, nitrate reductase, and sucrose phosphate synthase in vitro. Plant Physiol..

[40] Purcell P.C., Smith A.M., Halford N.G. (1998). Antisense expression of a sucrose nonfermenting-1-related protein kinase sequence in potato results in decreased expression of sucrose synthase in tubers and loss of sucrose-inducibility of sucrose synthase transcripts in leaves. Plant J..

[41] Polge C., Thomas M. (2007). SNF1/AMPK/SnRK1 kinases, global regulators at the heart of energy control. Trends Plant Sci..

[42] Ball S., Guan H., James M., Myers A., Keeling P., Mouille G., Buléon A., Colonna P., Preiss J. (1996). From glycogen to amylopectin: A model for the biogenesis of the plant starch granule. Cell.

[43] Zeeman S.C., Umemoto T., Lue W.L., Au-Yeung P., Martin C., Smith A.M., Chen J. (1998). A mutant of *Arabidopsis* lacking a chloroplastic isoamylase accumulates both starch and phytoglycogen. Plant Cell.

[44] Hussain H., Mant A., Seale R., Zeeman S., Hinchliffe E., Edwards A., Hylton C., Bornemann S., Smith A.M., Martin C. (2003). Three isoforms of isoamylase contribute different catalytic properties for the debranching of potato glucans. Plant Cell.

[45] Chang M., Zhu G., Liu K. (2026). Low-molecular-weight ferulic oligosaccharides derived from rice bran enhance the inhibitory effect on rice starch digestion by attenuating self-aggregation. Carbohydr. Polym..

[46] Yao G.F., Zhang S.L., Cao Y.F., Liu J., Wu J., Yuan J., Zhang H.P., Xiao C.C. (2010). Characteristics of components and contents of soluble sugars in pear fruits from different species. Sci. Agric. Sin..

